# An Investigation into the Effects of Processing Factors on the Properties and Scaling-Up Potential of Propranolol-Loaded Chitosan Nanogels

**DOI:** 10.3390/pharmaceutics16050662

**Published:** 2024-05-15

**Authors:** Hei Ming Kenneth Ho, Richard M. Day, Duncan Q. M. Craig

**Affiliations:** 1 School of Pharmacy, University College London, 29-39 Brunswick Square, London WC1N 1AX, UK; 2 Centre for Precision Healthcare, UCL Division of Medicine, University College London, 5 University Street, London WC1E 6JF, UK; 3 Faculty of Science, University of Bath, Claverton Down, Bath BA2 7AY, UK

**Keywords:** nanogels, stirring, processing factor, scaling up, optimisation

## Abstract

Chitosan-triphosphate (TPP) nanogels are widely studied drug delivery carrier systems, typically prepared via a simple mixing process. However, the effects of the processing factors on nanogel production have not been extensively explored, despite the importance of understanding and standardising such factors to allow upscaling and commercial usage. This study aims to systematically evaluate the effects of various fabrication and processing factors on the properties of nanogels using a Design of Experiment approach. Hydrodynamic size, polydispersity index (PDI), zeta potential, and encapsulation efficiency were determined as the dependent factors. The temperature, stirring rate, chitosan grade, crosslinker choice, and the interaction term between temperature and chitosan grade were found to have a significant effect on the particle size, whereas the effect of temperature and the addition rate of crosslinker on the PDI was also noteworthy. Moreover, the addition rate of the crosslinker and the volume of the reaction vessel were found to impact the encapsulation efficiency. The zeta potential of the nanogels was found to be governed by the chitosan grade. The optimal fabrication conditions for the development of medium molecular weight chitosan and TPP nanogels included the following: the addition rate for TPP solution was set at 2 mL/min, while the solution was then stirred at a temperature of 50 °C and a stirring speed of 600 rpm. The volume of the glass vial used was 28 mL, while the stirrer size was 20 mm. The second aim of the study was to evaluate the potential for scaling up the nanogels. Size and PDI were found to increase from 128 nm to 151 nm and from 0.232 to 0.267, respectively, when the volume of the reaction mixture was increased from 4 to 20 mL and other processing factors were kept unchanged. These results indicate that caution is required when scaling up as the nanogel properties may be significantly altered with an increasing production scale.

## 1. Introduction

Nanogels are nanosized particles composed of a network of solvated and cross-linked hydrophilic polymers. These systems have strong water-holding ability without self-dissolution or self-disintegration, thereby generating a three-dimensional hydrophilic matrix that allows encapsulation of active pharmaceutical ingredients. Amongst the nanogels reported in the literature, chitosan-triphosphate nanogels are one of the most extensively researched systems, being used for a variety of applications [1,2,3,4,5,6,7,8,9]. Chitosan is a linear polycationic polysaccharide derived from chitin found in the cell walls of fungi, exoskeletons of arthropods, and shells in crustaceans [10,11,12]; it is biocompatible, relatively inexpensive, non-toxic, and mucoadhesive [13], and is thus a suitable material for use as a drug delivery vehicle.

A variety of possible chemical modifications may be performed on chitosan, allowing optimisation of performance properties according to the physical and biological requirements [14,15], including polymer molecular weight, the degree and distribution of acetylation, and modification of the amine group. Moreover, as a polycationic polymer, it has the propensity to complex with genetic materials or other anionic peptides or proteins while facilitating intracellular uptake associated with other nano-carriers [16,17]. One gelling agent typically used with this polymer, sodium triphosphate (TPP) [2,18,19,20,21,22,23,24,25], is considered safer and thus preferable to more toxic alternatives such as glutaraldehyde, which is an irritant to the eyes, mucous layers, and skin. Moreover, TPP interacts with the chitosan spontaneously, thereby requiring relatively mild fabrication conditions to form nanogels via ionic gelation.

Chitosan is crosslinked via the positively charged amine groups and the negatively charged phosphate groups in TPP; while the associated electrostatic interactions are strong, there is a risk of precipitation if those interactions are excessive. Conversely, the 3D conformational structure may not be maintained in biological fluids if the interaction is too weak [26]. Therefore, optimal crosslinking between chitosan and TPP provides mechanical strength, compatibility, and durability to the nanogels. Indeed, a wide range of payloads, such as small-molecule drugs, photosensitizers, proteins, and genes, have been successfully delivered to cells and animals using chitosan-TPP nanogels as carriers [5,6,8,9,27,28,29,30]. Furthermore, these nanogels may also be modified by coating and surface modifications, offering a versatile architecture for different applications [4,31,32].

Several fabrication methods have been explored for chitosan-based nanogels, including stirring, micro-/nano-emulsion templating [33,34,35], self-assembly [36,37,38,39] and radical polymerisation [40,41]. Furthermore, a more advanced and controllable technique, microfluidics, may also be used in nanogel fabrication [28]. Despite being a continuous manufacturing process, fouling and blockage of the channel remain significant challenges in nanogel fabrication via microfluidics. Meanwhile, stirring is by far the simplest and most extensively used method for fabricating nanogels [1,2,20,21,42,43]. However, the mechanisms associated with the mixing and the effect of the processing factors are not well explored in the literature and were thus investigated in this study.

**Figure 1:**
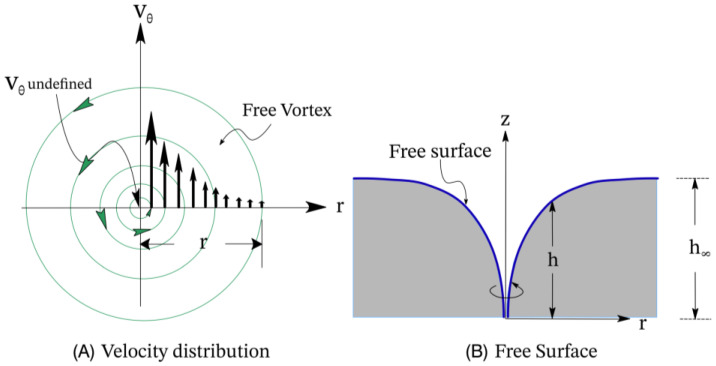
Illustration showing (**A**) the velocity distribution and (**B**) the free surface of the vortex in the Rankine model (adapted from [46]. Copyright 2019 by Elsevier).

In general, nanogel fabrication via mixing is governed by a combination of mixing and gelation processes. Chitosan solutions are typically viscous, with this being dependent on the molecular weight and the concentration used. A vortex is generated by the rotation of a solid body (i.e., a magnetic bead) within the reaction vessel; hence, the mixing process may be described by the Rankine Vortex model, which is a simple mathematical model of a vortex in a viscous fluid [44]. In this model, there are two different flow regions: a rigid inner core vortex and a free (irrotational) vortex, as shown in Figure 1. The radius R is the radius of the stirrer, while the radial distance r denotes the distance from the vortex core, with the maximum equivalent to the radius of the cylindrical container. V_θ_ is the tangential velocity of the flow. In the inner core, where the radial distance r < R, rigid body rotation takes place, which indicates that the motion resembles the solid-body motion (i.e., rotation) in the core vortex. Meanwhile, efficient mixing occurred in the free vortex (r > R), where the tangential velocity correlates inversely with the radius r. However, there is a breaking point between the core vortex and the free vortex at r = R in the model [45]. The model is simple and capable of accurately describing the geometry of the vortex in a 2D manner, but the stirring of solutions happens in a 3D space in reality. Thus, the flow also exhibits a toroidal motion, where a downward jet is formed on the axis of the vortex and upward streams are at the outer walls [44]. The toroidal flow also helps mix the solutions on the vertical axis-*z*.

The second important process is gelation, which is governed by the ionic interaction between chitosan and crosslinkers and may be considered a nucleation process. When the two components come into proximity via the mixing process, the gelation process will be initiated, leading to nucleation [47]. Combining the Rankine model with the gelation process, it is known that the velocity decays with the distance from the edge of the rotating object in the free vortex. Therefore, the gelation is likely to take place in this free vortex region, as chitosan and TPP may diffuse and interact. At a microscopic level, the mixing process is also controlled by diffusion, as the chitosan and the crosslinker’s movements are governed by the concentration gradient. Thus, the diffusivities of these components are also crucial in the process, which in turn is dependent on the temperature and the dynamic viscosity of the solution according to the Stoke-Einstein equation—Equation (1):

(1) $$D=\frac{K_{B}T}{6\pi \eta r}$$

where D is the diffusion coefficient, K_B_ is Boltzmann’s constant, *T* is the temperature, *η* is the dynamic viscosity, and *r* is the radius of the spherical particle.

There are four key properties to be controlled in a nanogel system, namely hydrodynamic size (size), polydispersity index (PDI), zeta potential (ZP), and encapsulation efficiency (EE); these are related to the drug delivery efficiency, stability, and ability to encapsulate payloads. The target properties for the nanogels—size < 200 nm, PDI < 0.3, ZP > 30 mV, and EE > 50% were chosen for this study. It is hypothesized that the optimisation of the fabrication process is crucial in controlling nanogel properties. Nine processing factors were tested in this study, and given the theoretical considerations outlined above, it is reasonable to propose that they may influence mixing and gelation. More specifically, the radius R and maximum radius r in the Rankine model are controlled by the stirrer size (SS) and glass vial volume (GV), respectively, while the total volume of liquid (TV) impacts the vortex depth and subsequently the toroidal flow. Furthermore, stirring rate (SR) determines the linear velocity V_θ_ and subsequently the Reynold number, where the latter is a function of the reaction rate constant *k* [48]. Mixing time is defined as the time required to achieve homogeneity in the stirred vessel [49]. Hence, the duration of stirring (D) could only affect the homogeneity of the stirred solution when insufficient time was allowed to mix. Meanwhile, temperature (T) and addition rate (AR) have an important role in diffusion, impacting the diffusivity and concentration gradient, respectively. Moreover, the use of different chitosan grades and TPP analogues was also explored, as both factors could alter the electrostatic interactions and the viscosity, subsequently affecting both diffusion and nucleation.

Overall, this study aims to evaluate the effects of these processing factors systematically via a Design of Experiment approach and to identify an optimised condition for nanogel fabrication. Propranolol was used as the model drug as it has good chemical stability, while its pharmacological effects are such that nanogel formulations may have therapeutic advantages, a possibility explored in a separate study. The formulation was kept unchanged to minimize variations in the nanogel properties [50]. Finally, after the fabrication process was optimised, the scalability of the method in terms of the volume of nanogels fabricated in each vessel was investigated, as such scalability represents a highly important consideration in subsequent product development and manufacture.

## 2. Materials and Methods

### 2.1. Materials

Low molecular weight (LMW) and medium molecular weight (MMW) chitosans were purchased from Sigma Aldrich (St. Louise, MO, USA) with a molecular weight range of 50–190 and 190–310 kDa, respectively, according to the manufacturer. Sodium pyrophosphate was also acquired from Sigma Aldrich. Penta-basic sodium triphosphate (TPP) was purchased from Fluka (Buchs, Switzerland), while propranolol hydrochloride (propranolol) was acquired from Acros Organics (Geel, Belgium). Glacial acetic acid was obtained from Fisher Scientific (Waltham, MA, USA). Sodium hydroxide pellets were acquired from VWR (Radnor, PA, USA). All chemicals were at analytic grade and used as supplied. Deionised water was obtained from a PURELAB^®^ Chorus 2+ machine (ELGA LabWater, High Wycombe, UK). Stirrers with three assorted sizes and two glass vial volumes were used in the DSD, with the dimensions (measured using a calliper) shown in Table 1.

### 2.2. Nanogel Fabrications

Propranolol-loaded chitosan nanogels were prepared according to a previously developed method and formulation [50]; the formulation had been previously optimised and thus used without modification in this study. Low- and medium-molecular weight chitosans were first dissolved in a 1% acetic acid solution until they formed a clear solution, followed by pH adjustment to pH 4.5 with a 0.1 M sodium hydroxide solution. The final concentration of chitosan was 1 mg/mL. Propranolol hydrochloride was weighed and subsequently dissolved into the chitosan solution to achieve 2 mg/mL. Meanwhile, TPP was dissolved in deionised water at 0.33 mg/mL. All solutions were filtered with a 0.22 µm syringe filter before use (Millipore, Darmstadt, Germany). An equal amount of TPP solution was added to the chitosan under constant stirring at 600 rpm for 1 h on a digital multi-position magnetic hotplate stirrer (RT 10; IKA, Staufen im Breisgau, Germany), where the temperature was controlled during the fabrication process. Nanogels were prepared in triplicate and then kept in a fridge at 4 °C for further characterisation the next day.

### 2.3. Screening the Effects of Parameters in the Fabrication Process

**Figure 2:**
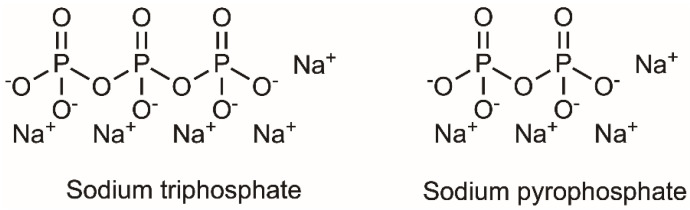
Structure of the phosphate−containing crosslinkers used in the fabrication of the nanogels.

To evaluate the effects of processing factors on the fabrication outcome, Z-average (size), polydispersity index (PDI), zeta potential (ZP), and encapsulation efficiency (EE), a definitive screening study (DSD) was used, where seven parameters were screened in the study, namely temperature (T), total volume (TV), stirring speed (SR), the addition rate of TPP solution (AR), duration of stirring (D), glass vial size (GV), and size of the magnetic stirrer (SS). As these factors are continuous except for the glass volume, they were modelled in three levels. Glass volume was considered a categorical factor in this study, as the volume of glass vials was fixed at 14 and 28 mL. Moreover, three additional factors were included in the design, namely blocking (B), chitosan grade (CS), and choice of crosslinkers (CR), to evaluate whether these factors affect the properties of nanogels. In this study, blocking was used to balance out the effect of inter-day variations on the fabrication, whereby nanogels in each block were fabricated on a separate day. Moreover, the chitosan grade and choice of crosslinkers were added to the study to evaluate the effect of different material attributes on the properties of nanogels. As chitosan is a natural polymer, its properties, including solubility and charge density, vary between manufacturers, batches, grades, origin, molecular weight, and the degree of deacetylation. Therefore, it is important to understand the effect on nanogel properties when different chitosans are used. To simplify the variations in chitosan, two batches of chitosan were purchased from the same manufacturer and were classified as different chitosan grades based on the molecular weights—LMW and MMW. It is noted that while the molecular weight of chitosan changes when a different grade is used, the degree of deacetylation also varies. Thus, the observed effects on nanogel properties may not be solely related to the molecular weight of chitosan. Regarding the choice of the crosslinkers, only two crosslinkers were used in this study, with their structures shown in Figure 2. Apart from TPP, a smaller analogue, sodium pyrophosphate (Pyro), was used, where pyrophosphate is a synonym for diphosphate. Therefore, it would be interesting to evaluate if these structurally similar crosslinkers interacted with chitosan comparably or not. Blocking, glass vial size, chitosan grade, and choice of crosslinker belonged to categorical factors, and thus they were modelled at two levels only.

A total of twenty-six experimental runs were performed according to the composite matrix shown in Table 2 in triplicate to construct the definitive screening design (DSD), where the composite matrix was constructed using JMP 15 (SAS Institute, Cary, NC, USA). The definitive screening design was then fitted by the Effective Model Selection for DSDs methodology, which was performed automatically in JMP 15. The main effects and second-order effects were first estimated individually and then combined to form the model parameter estimates.

The identified factors were then used in the optimisation step. A stepwise least squares regression was used to fit the polynomial model to the data individually for each dependent variable. Five-fold cross-validation was performed to validate the model for all dependent variables. A one-way analysis of variation (ANOVA) test and a lack of fit test were conducted to determine the statistical significance and goodness of fit for the model, respectively, at a confidence interval (CI) of 95%. Response surfaces were plotted to visualise the relationship between independent and dependent variables. A *p*-value < 0.05 is considered statistically significant.

### 2.4. Multiple Response Optimisation

Multiple response optimisation was used to determine the optimal fabricating condition for propranolol-loaded nanogels, as the dependent variables might counteract each other. This approach transformed the response variables (*y_n_*) into an individual desirability function *d_n_(y_n_)*, with a number assigned between 0 and 1. *d_n_(y_n_)* = 0 indicates a completely undesirable response, while *d_n_(y_n_)* = 1 represents the most desirable response. Individual desirability functions were transformed using JMP 15 software to minimise the particle size and PDI while maximising the EE and ZP. Individual desirability functions were then combined into overall desirability, as shown in Equation (2).

(2) $$D=\sqrt[{n}]{(d_{1}\left(y_{1}\right)\times d_{2}\left(y_{2}\right)\times \ldots \times d_{n}\left(y_{n}\right)},$$

where *d*_1_*(y*_1_*)* and *d*_2_*(y*_2_*)* denote the individual desirability function for factors 1 and 2, respectively. *n* is the total number of factors, and *d_n_(y_n_)* is the individual desirability function of factor *n*.

The running conditions with the highest overall desirability were deemed the optimal conditions and were determined by JMP 15. Nanogels were then fabricated under the optimal conditions in triplicate, with the dependent variables measured experimentally and compared with the predicted values to validate the models. The nanogels produced were then freeze-dried and characterised.

### 2.5. Characterisation Techniques for Nanogels

#### 2.5.1. Transmission Electron Microscopy

The shape and morphology of the nanogels were characterised using an FEI CM120 Bio Twin Transmission Electron Microscope (TEM) (Hillsboro, OR, USA). One drop of the nanogel sample was dropped onto 200-mesh carbon lacey-coated copper grids and stained with a 1% uranyl acetate solution, followed by air-drying at room temperature for a few minutes. The excess solution was removed using filter paper. Particle size distribution was performed using Image J 1.54h (NIH, Bethesda, MA, USA).

#### 2.5.2. Dynamic Light Scattering (DLS) and Electrophoretic Light Scattering (ELS)

The Z-average particle size and polydispersity of the nanogels were measured with a Zetasizer Ultra (Malvern Panalyticals, Malvern, UK) at room temperature using a backscatter angle of 173°. A disposable polystyrene cuvette was employed in the analysis. Zeta potentials were measured using U-shaped capillary cells (DTS 1070, Malvern Panalyticals, Malvern, UK) on the same machine. The results were measured in triplicate and obtained from three independent experiments.

#### 2.5.3. Encapsulation Efficiency of Propranolol in Chitosan-TPP Nanogels

The measurement of the EE of propranolol was adapted from the method reported by Al-Kassas et al. [29]. A total of 0.5 mL of the propranolol-loaded nanogel solutions were loaded into a 0.5 mL Amicon Ultra diafiltration tube (MWCO 3000; Merck Milipore, Billerica, MA, USA). The solutions were then centrifuged at 14,000× *g* for 30 min at 4 °C using a refrigerated mini centrifuge (Heraeus Fresco 17, Thermo Scientific, Waltham, MA, USA), and the filtrate was isolated and assayed by a UV-Vis spectrometer (Jenway 6305, Vernon Hills, IL, USA). The wavelength was set at 280 nm, and drug concentrations were calculated using a pre-determined calibration curve. EE% was calculated using Equation (3). The experiment was repeated three times, and the results were presented as mean ± SD.

(3) $$EE\%=\frac{D_{Theoretical}-D_{Free}}{D_{Theoretical}}\times 100\%,$$

where *D_Theoretical_* refers to the amount of propranolol added to the solution while *D_Free_* refers to the amount of propranolol present in the aliquot after centrifugation.

### 2.6. Fourier Transform Infrared Spectroscopy (FTIR)

Analysis was performed with a Spectrum 100 FTIR spectrometer equipped with an attenuated total reflectance (ATR) sampling accessory (Perkin Elmer, Waltham, MA, USA) in the range of 650*–*4000 cm*^−^*^1^ and with a resolution of 1 cm*^−^*^1^.

### 2.7. Scaling up of the Nanogel Fabrication

Nanogels were fabricated at 4 different total volumes ranging from 2 to 20 mL at the optimal fabrication conditions and formulation identified above. The total volume is defined as the sum of volumes for chitosan solution and TPP solution. A total of 4 mL was used as the total volume (i.e., 2 mL of chitosan solution mixed with 2 mL of TPP solution) in the optimisation studies described above and thus was used as the control. A one-way ANOVA test with Dunnett’s post hoc test was used to ascertain if the properties of nanogels at different volumes were different from the control.

## 3. Results

### 3.1. Definitive Screening Design (DSD)

**Figure 3:**
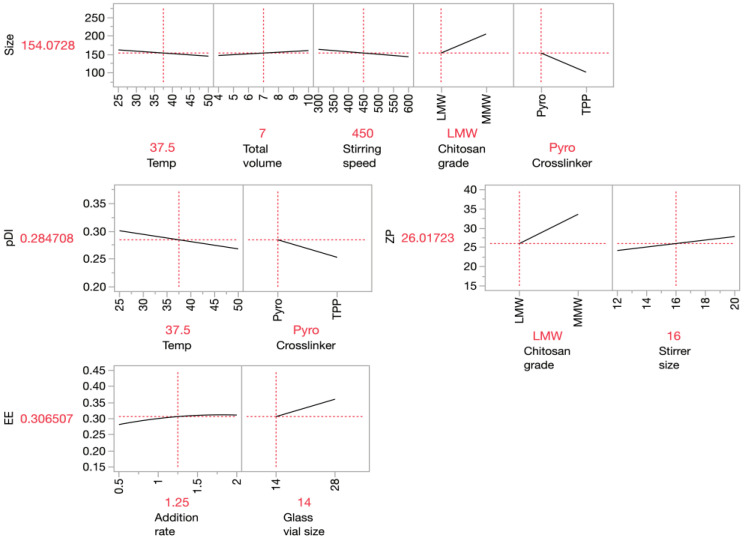
Profilers show the effect of the factors identified from the DSD on each independent variable.

The factors with linear effects were first screened in the definitive screening design for each nanogel property (size, PDI, ZP, and EE), followed by the factors with quadratic and interaction effects. The processing parameters were estimated to have effects on nanogel properties using the profilers shown in Figure 3. Temperature, total volume of the solution, stirring speed, grade of chitosan, and choice of crosslinkers were estimated to impact the size of the nanogels, while temperature and choice of crosslinker also influenced the PDI of nanogels. Furthermore, ZP was estimated to be dependent on the chitosan grade and, interestingly, the size of the stirrer. Nevertheless, the addition rate of the crosslinker and glass vial volume was expected to affect the encapsulation efficiency of the nanogels. These processing factors were selected to construct a model for the respective nanogel properties.

### 3.2. Response Surface Methodology

The selected parameters were used to construct a model via stepwise regression, with 5-fold cross-validation. For size, the model became saturated when all terms were included. Thus, terms with the highest *p*-value were removed sequentially until the model was not saturated to avoid overfitting and improve the effectiveness and robustness of the approach. Two interactions (T × CR and T × SR) and total volume (TV) were therefore removed from the model. A one-way analysis of variation (ANOVA) and a lack of fit test were then performed on the linear regression models for each individual dependent variable to determine the statistical significance and the goodness of fit of these models on the training set. The null hypothesis of the ANOVA is that the nanogel properties do not correlate with the parameters. The results of the ANOVA and lack of fit tests are reported in Table 3. The *p*-values obtained in the ANOVA test for all the models were smaller than 0.05, demonstrating the significance of the correlations between the training set and the models. Furthermore, the *p*-values in the lack of fit tests for all models were larger than 0.05, which indicates these models were a good fit for the training set data. However, it should be noted that a good fit with the training data do not necessarily indicate good predictability; a further test set is usually performed for such a purpose but was not performed here as the current study is focused on optimisation rather than predictability. In short, the results indicated that the models were well-fitted to the data set and the correlations between the processing factors and the properties of nanogels were significant. Hence, an optimal set of conditions was obtained from the multiple response optimisation.

### 3.3. Effect of Processing Factors on Nanogel Properties

#### 3.3.1. Z-Average

**Figure 4:**
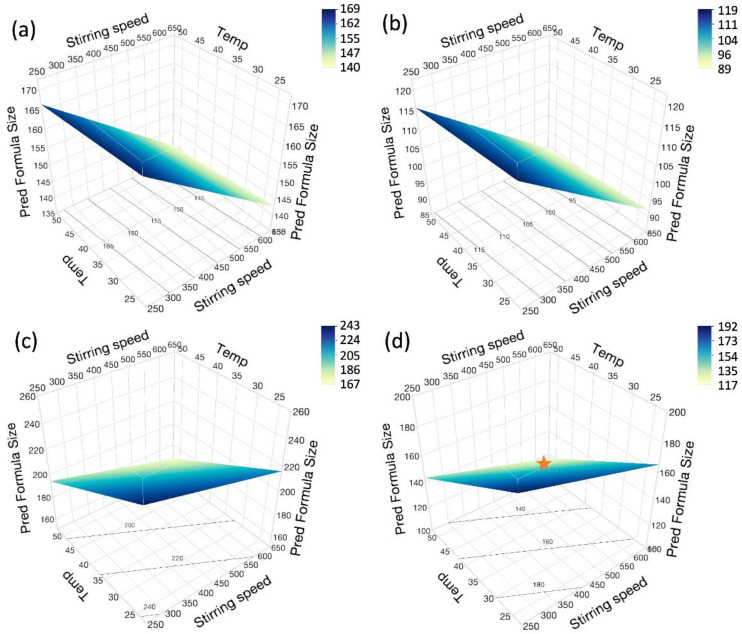
Response surface models predicting the effect of temperature and stirring rate on the size of the nanogels at four different combinations of chitosan grade and crosslinkers, as the response surface plots, could show the continuous factors only. Thus, the surface plots were presented in 4 combinations, namely (**a**) LMW chitosan and pyro, (**b**) LMW chitosan and TPP, (**c**) MMW chitosan and pyro, and (**d**) MMW chitosan and TPP. The optimal condition was indicated as the orange star on the surface plot (**d**).

In the definitive screening design, temperature, stirring rate, grade of chitosan, and choice of crosslinker were found to be important factors in the Z-average of nanogels, as presented in Table 3. In particular, the size of the nanogels decreased with stirring speed, as shown in Figure 4a. As the reaction rate constant increased with the stirring speed, the mixing of the two solutions improved, resulting in a higher probability of chitosan interacting with the crosslinker. Thus, more nucleation sites and, hence, smaller particles were formed. Similar results were observed by Hussain et al. for this stirring speed range, where the particle size of chitosan TPP nanoparticles decreased with the stirring speed varying from 200 to 700 rpm [51]. However, this group also found that the effect of stirring speed on the nanoparticle size was a V-shape or quadratic effect. Hence, the size of nanoparticles increased when the stirring speed was further increased from 700 rpm to 1000 rpm. This observation was likely due to the poor mixing effect when the vortex was too deep and reached the impeller at high stirring speed.

The choice of crosslinkers was also found to influence the size, with nanogels formed with pyrophosphate being larger than those formed by TPP. This may be due to differences in the nature of the linking chemistry between chitosan and the crosslinkers. Pyrophosphate is a diphosphate carrying only four negative charges, while TPP refers to triphosphate, which bears five negative charges. Hence, the electrostatic interactions between chitosan and pyrophosphate are weaker than those between chitosan and TPP. Consequently, the nanogels were less contracted due to electrostatic interaction. In addition, the conformational coordination of the crosslinkers was thought to be different between the pyrophosphate and TPP, where a TPP molecule arranged in a V-shape interacted with the amine group on chitosan to form two crosslinks per molecule. In contrast, pyrophosphate interacted with anime groups on chitosan and formed one crosslink per molecule [52]. Furthermore, chitosan grade was found to impact the size, with LMW chitosan producing smaller nanogels than MMW chitosan. This may be related to the higher viscosity of the MMW chitosan solution, resulting in superior mixing for the LMW system, in turn leading to smaller nanogels. Similarly, at high temperatures, the viscosity of the chitosan solution is reduced, again improving diffusivity and mixing, leading to smaller nanogels. Nevertheless, there was also a synergistic effect between temperature and the chitosan grade on particle size. As an overarching finding, the mathematical expression of the correlations of the processing factors and the Z-average is described by Equation (4).

(4) $$SIZE=216.8-0.069SR-0.863\times T-3\times CS\left(\begin{array}{cc} LMW & 9.517\\ MMW & -9.517 \end{array}\right)\;+0.08\times T\times CS\left(\begin{array}{cc} LMW & 9.517\\ MMW & -9.517 \end{array}\right)+CS\left(\begin{array}{cc} LMW & -25.209\\ MMW & 25.209 \end{array}\right)\;+CR\left(\begin{array}{cc} Pyro & 25.357\\ TPP & -25.357 \end{array}\right),$$

In summary, the fabrication process is critical to controlling the hydrodynamic size of nanogels. Temperature and stirring speed demonstrated an inverse correlation with size. Moreover, the particle size of the nanogels was smaller when LMW chitosan and TPP were used. The results highlight the difficulties in reliably replicating the nanogel production process without knowing the details of the manufacturing parameters used for the nanogel fabrication.

#### 3.3.2. PDI

From a pharmaceutical perspective, the population of the nanocarriers should be as homogenous as possible. The PDI is a measure of the homogeneity of the nanoparticles in terms of size distribution [53], indicated by a value between 0 and 1 for the Malvern Zetasizer series. Hence, the smaller the PDI, the more uniform the size of the nanogels. A high PDI value (>0.7) denotes a very broad size distribution of the nanoparticles, which might indicate agglomeration of the nanoparticles or the presence of other contaminants.

The PDI values of the nanogels shown in Table 2 were between 0.218 and 0.355, indicating that all formulations were moderately dispersed. Only temperature and crosslinker choice were the processing factors found to affect the PDI of the nanogels, as shown in Equation (5). At high temperatures, the mixing was improved as the viscosity of the chitosan solution and crosslinker solution was lowered. Therefore, the particles were likely more homogenous in size, and thus the particle size distribution was narrower, while the PDI decreased at high temperatures. Moreover, the choice of crosslinkers was equally important, with higher polydispersity obtained with pyrophosphate than with sodium triphosphate.

(5) $$PDI=0.318-0.001\times T+CR\left(\begin{array}{cc} Pyro & 0.016\\ TPP & -0.016 \end{array}\right),$$

#### 3.3.3. Zeta Potential

The propranolol-loaded nanogels are formed by ionic gelation between cationic chitosan and anionic crosslinkers, where chitosan is used in 3-fold of the crosslinkers. Therefore, nanogels are generally positively charged in acidic conditions with a pH < 6, where the amine groups on the chitosan are protonated. The ZP of nanogels is an important influencer on the colloidal stability of the nanogels, as the agglomeration of nanogels is attenuated by electronic repulsion [54,55]. Nanogels with ZP values of 30 mV are generally stable in suspension due to the presence of sufficient electronic repulsion between particles [56]. The zeta potentials of the nanogels from the data set, as shown in Table 2, were in the range of 17 to 38 mV, which indicated that around half of the nanogel formulations were stable due to the surface charge in the suspension.

Only the chitosan grade was found to have a significant effect on the ZP of the drug-loaded nanogels, as shown in Equation (6); other parameters were found not to have a statistically significant influence on this property. The caveat of the study is that, being a natural polymer, two different grades of chitosan with identical molecular weight or degree of deacetylation could not be purchased, and thus two grades of chitosan with different molecular weight and degree of deacetylation were used. Therefore, the higher ZP in nanogels fabricated with MMW chitosan may be due to the higher molecular weight, degree of deacetylation, or a combination of both. The degree of deacetylation indicates the proportion of free amine groups on the chitosan, which would be protonated at the fabricating pH. Hence, MMW chitosan had more free amine groups in the chain and was subsequently more cationic than LMW chitosan at pH 4.5. Thus, the surface charge was neutralised by the anionic crosslinker to a lesser degree, resulting in higher ZP.

(6) $$ZP=22.617+0.454\times SS+CS\left(\begin{array}{cc} LMW & -3.805\\ MMW & 3.805 \end{array}\right),$$

In summary, only the grade of chitosan was found to have a significant effect on ZP, where higher zeta potential was obtained in propranolol-loaded nanogels fabricated with MMW chitosan. However, this dependence could be due to the higher molecular weight, the higher degree of deacetylation, or a combination of both.

#### 3.3.4. Encapsulation Efficiency

Only two processing factors, the addition rate of crosslinker solution and glass vial volume, were found to have significant effects on the EE as present in Equation (7), with higher EE observed at a higher addition rate and when a larger container was used. This may be due to the faster addition rate leading to more rapid TPP or pyrophosphate availability for cross-linking. Hence, the gelation process was faster, and more propranolol could interact with the TPP or pyrophosphate at the beginning of the gelation stage, which enhanced the encapsulation efficiency in the nanogels. Interestingly, the encapsulation efficiency was also dependent on the glass vial volume, with nanogels fabricated in the larger glass vial (volume = 28 mL), possessing higher encapsulation efficiency. Glass vial volume was considered a categorical data set, as the radius and height of the glass vial changed simultaneously for the larger glass vials, and no intermediate size was found between the two vial sizes. The higher encapsulation efficiency using a larger vial is probably related to the vortex mixing, where the radius (r) of the container plays an important role. The velocity of the flow decreased with 1/r^2^ outside the vortex. The larger the radius of the vial, the slower the velocity, and thus more time was available for propranolol to interact with the crosslinkers and encapsulate during the gelation process.

(7) $$EE=0.295+0.024\times AR+GV\left(\begin{array}{cc} 14 & -0.027\\ 28 & 0.027 \end{array}\right)$$

### 3.4. Multiple Response Optimisation

**Figure 5:**
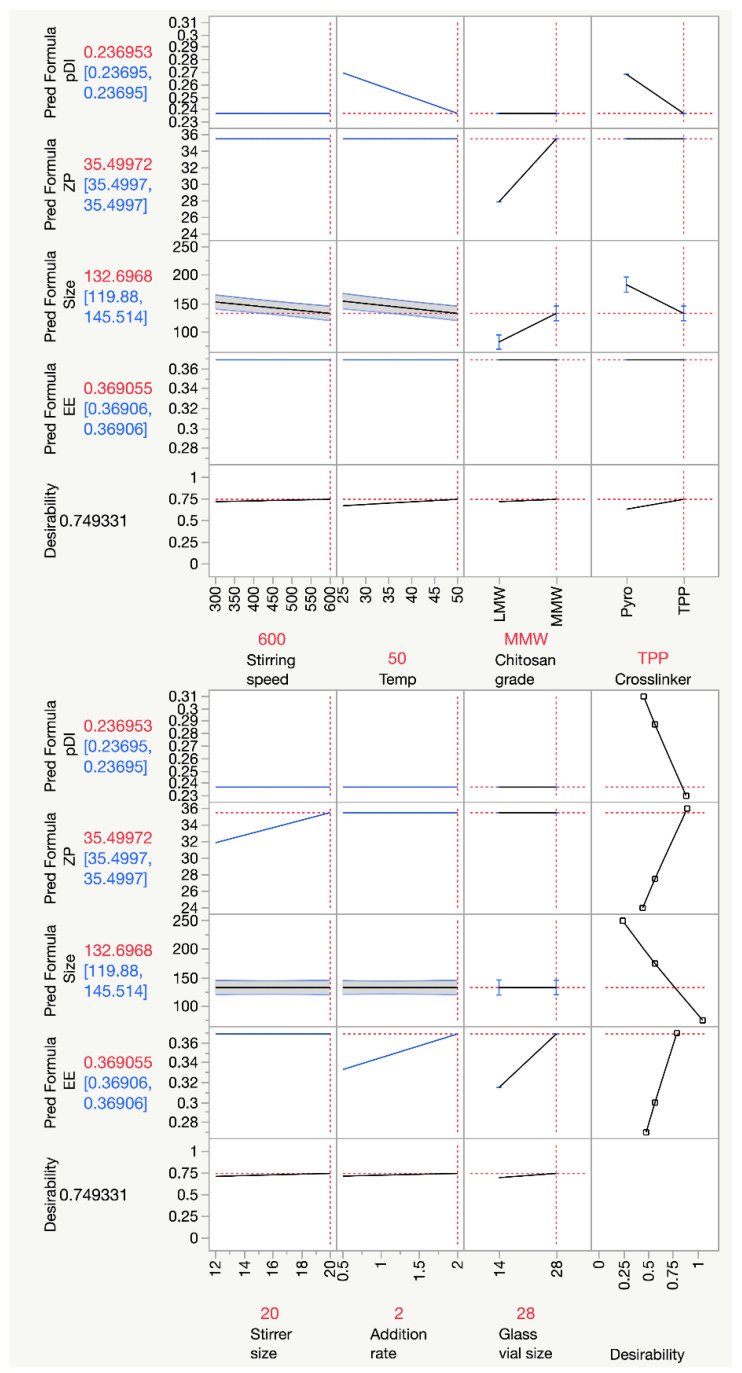
The prediction tool shows the relationship between the parameters the outcome and the optimal condition for nanogel fabrications.

Both screening and response surface methodologies could be performed with a single model of definitive screening design. Thus, an optimal fabricating condition could also be determined by multiple response optimisation (MRO) based on the established correlations. The MRO aimed to maximise EE and ZP and lower Z-average and PDI. The profilers in Figure 5 summarised all correlations between nanogel properties and the processing factors. A horizontal line in the profiler indicated no correlation between the nanogel properties and the respective processing factors, whereas a slope indicated a linear relationship. Each response was converted into a desirability function ranging between 0 and 1, as discussed in Section 2.4. They were shown in the last column in Figure 5 for each response. The optimal fabrication for nanogel production was shown in red on the *x*-axis, and the overall desirability of the optimal condition was shown as 0.749. The optimal condition utilised MMW chitosan and TPP, with an additional rate for TPP solution at 2 mL/min. The solution was then stirred at a temperature of 50 °C and a stirring speed of 600 rpm. The volume of the glass vial used was 28 mL, while the stirrer size was 20 mm. The predicted properties of nanogels fabricated at this condition were presented in red and shown on the *y*-axis, which were 133 nm, 0.237, 35.5 mV, and 36.9% for the predicted size, PDI, ZP, and EE, respectively. However, the measured results of the nanogels were 114 ± 6 nm, 0.215 ± 0.009, 20.9 ± 7.2 mV, and 58.9 ± 5.2%, which were −16.8%, −10.2%, −69.9%, and 59.6% different from the predicted values, respectively. The nanogels fabricated fulfilled most of the target criteria set in this study, except ZP. The high discrepancies between the measured and predicted values for ZP and EE indicated that other factors that were not included in the models impacted these properties, even though multiple processing factors were tested. Alternatively, the results might demonstrate that the encapsulation efficiency and zeta potential could not be effectively predicted and were partly random. In short, an optimal fabrication condition was obtained from the DSD, with the size and PDI of nanogels being effectively predicted.

Overall, despite using the same formulation, the properties of nanogels are greatly impacted by the fabrication process. Thus, it is crucial to consider the fabrication process when optimising the nanogels. It also illustrated the difficulties in replicating the nanogels to obtain similar properties, especially from a reported method in the literature, as the fabrication process parameters may be different and not reported. In particular, as chitosan is a natural polymer that could vary in molecular weight and degree of deacetylation even from the same manufacturer, it is likely that there would be batch-to-batch variation, which could add complexity to optimising nanogel formulations. Apart from chitosan grade and crosslinker choice, temperature, stirring speed, the addition rate of crosslinkers, glass vial size, and stirrer size also influence the properties and thus should be controlled and reported in the literature.

### 3.5. Characterisation Techniques for Raw Materials and Freeze-Dried Nanogels

#### 3.5.1. Fourier Transform Infrared Spectroscopy

**Figure 6:**
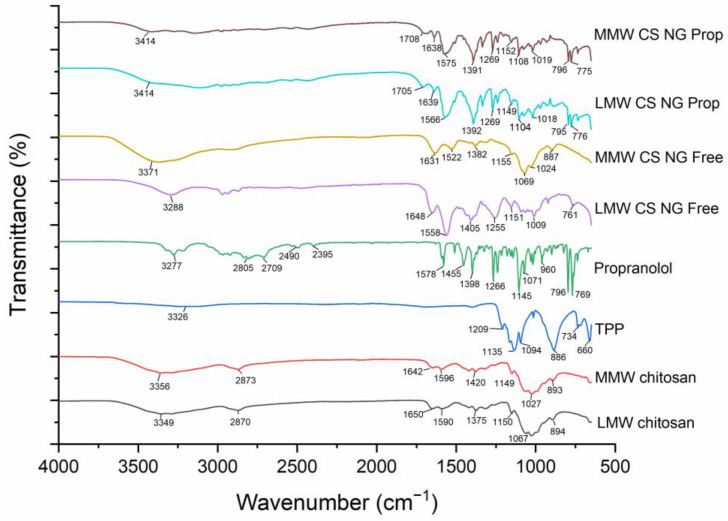
FTIR spectrum showing the components of the formulation individually, freeze-dried unloaded nanogels and the propranolol-loaded nanogels.

Figure 6 shows the IR spectrum of the individual components of the nanogels, as well as both propranolol-loaded and drug-free nanogels fabricated with LMW and MMW chitosan. In the spectra of LMW chitosan, a strong band at 3349 cm^−1^ is associated with O-H stretching with intramolecular hydrogen bonds, while the peak at 2870 cm^−1^ corresponds to asymmetric C-H stretching. Slight shifts in peaks were observed in the MMW chitosan. Similar bands at 3288, 3371, and 3414 cm^−1^ were observed in the freeze-dried chitosan nanogels, which also correspond to these intramolecular hydrogen bonds. The symmetric C-H stretching was not obvious in the spectra, as no peak was observed around 2900 cm^−1^. N-acetylation of chitosan was confirmed with the bands at 1642–1650 cm^−1^ and 1375–1420 cm^−1^, which are the C=O stretching of amide and C-N stretching of amide, respectively, as well as the peaks at around 1590 and 1596 cm^−1^, which correspond to the N-H bending. The strong peaks at 1027 and 1067 cm^−1^ are associated with the C-O stretching. The spectra agree with the results reported in the literature [57,58].

For propranolol, a band at 3277 cm^−1^ corresponds to the O-H stretching with intramolecular hydrogen bonds, but the N-H stretching with intramolecular hydrogen bonds was less obvious at about 3221 cm^−1^ in the spectrum. A peak at 796 cm^−1^ corresponds to the naphthalene in propranolol, while the aryl alkyl ether is associated with the peak at 1266 cm^−1^ [59]. C=C stretching in naphthalene is observed with a sharp peak at 1578 cm^−1^. The spectrum obtained agrees with other literature [60]. A board and shallow band at 3326 cm^−1^ in the spectrum of TPP corresponds to the O-H stretching, while another board peak at 1135 and 1209 cm^−1^ is associated with O-P=O and P=O stretching, respectively [61]. A sharp peak at 1094 cm^−1^ corresponds to P-O stretching. The sharp peaks at 1255 and 1269 cm^−1^ were present in the drug-free and propranolol-loaded nanogels fabricated with LMW chitosan, respectively, which are indicative of the P=O bond in TPP within the nanogel structure, albeit shifted from 1209 cm^−1^ in TPP alone as a result of the interaction with chitosan [28]. However, the peak was not observed in the propranolol-loaded nanogels fabricated with MMW chitosan. Drug-free nanogels fabricated with LMW chitosan exhibited sharper peaks at 1558 and 1648 cm^−1^ compared to the LMW chitosan, which showed that the complexation of LMW chitosan with TPP is likely to influence the chemical interaction between chitosan. In contrast, the peaks at 1522 and 1638 cm^−1^ in drug-free nanogels fabricated with MMW chitosan were less sharp than their counterparts fabricated with LMW chitosan, which illustrated that the complexation between MMW chitosan and TPP was less strong compared to LMW chitosan and TPP. Moreover, the C-O stretching of either group in chitosan was observed at 1087 cm^−1^, which shifted to 1009–1069 and 1018–1019 cm^−1^ in the drug-free and propranolol-loaded nanogels, respectively. The shift was similar to the reported literature [28]. Several distinct peaks for propranolol at 776, 795, and 1269 cm^−1^ were present in propranolol-loaded nanogels, which were not observed in the drug-free nanogels. In conclusion, the IR spectrum confirms the presence of the individual components in the nanogels and structural change of the nanogels after encapsulation and loading of propranolol was not observed.

#### 3.5.2. Transmission Electron Microscopy

**Figure 7:**
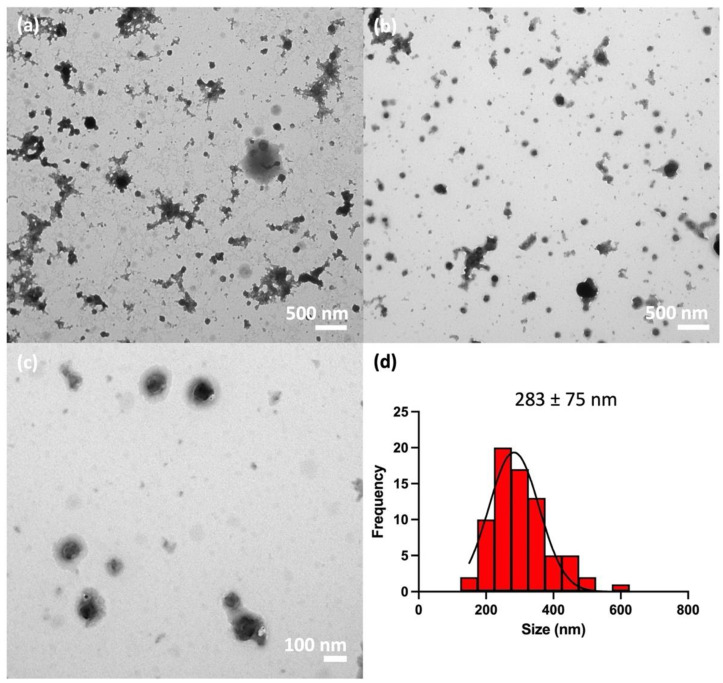
(**a**–**c**) TEM images of the propranolol-loaded nanogels fabricated with MMW chitosan and TPP at the optimal fabrication condition determined from the DSD and (**d**) corresponding particle size distribution based on the TEM images.

TEM images of the propranolol-loaded nanogels fabricated with MMW chitosan and TPP at the optimal fabrication condition are shown in Figure 7a–c, while the corresponding size distribution is shown in Figure 7d. The nanogels appeared as spherical or oval objects. The average size of the propranolol-loaded nanogels at the dried state was 283 ± 75 nm, of which the size distribution was calculated from 75 particles in three TEM images. The result was much larger than the measured Z-average of 114 ± 6 nm in the DLS. The discrepancy between the two values was likely to have occurred because the size measured in the TEM image was the diameter at the dry state, while the size measured with Zetasizer was the hydrodynamic size, where particles were suspended in water with an assumption of spherical particles. The wide particle size distribution in the TEM images demonstrated that the nanogels in the TEM samples were not sufficiently dried, and thus some nanogels may appear larger than others [28]. Paradoxically, the drying of the nanogels can also promote aggregate formation, further leading to the observation of large particles in the TEM images.

### 3.6. Scaling-Up of Nanogel Fabrication

**Figure 8:**
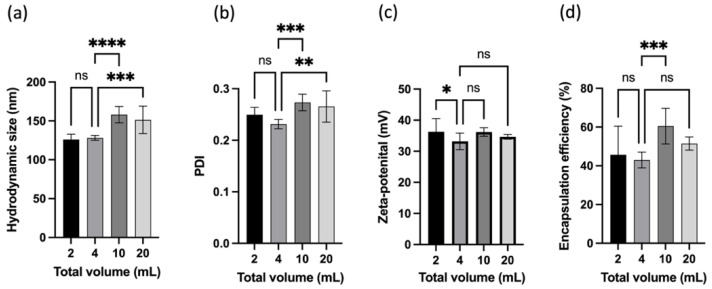
Properties of nanogels (**a**) size, (**b**) PDI, (**c**) zeta potential, and (**d**) encapsulation efficiency fabricated with different total volumes at the optimal formulation and fabrication condition. ns denoted not significant, * referred to *p* < 0.05, ** *p* < 0.01, *** *p* < 0.001 while **** *p* < 0.0001.

After determining the formulation and fabrication conditions, the scaling-up process of the nanogels was investigated. The ideal scenario would be for the nanogel manufacturing to be scaled up by increasing the volume pro rata. However, as the total volume was screened in the DSD but removed to prevent overfitting of the models, it may be anticipated that the total volumes might affect the properties of nanogels; hence, this factor was evaluated separately. The optimal fabrication conditions and formulation identified above were used to fabricate the nanogels. Four total volumes ranging from 2 to 20 mL were used, whereby total volume refers to the sum of volumes for chitosan solution and TPP solution in a 1:1 ratio, noting that 4 mL was used in all studies above. The size, PDI, ZP, and EE of the nanogels were measured and shown in Figure 8.

A one-way ANOVA with Dunnett’s multiple comparison tests was performed to determine the difference in the nanogel properties when the total volume varied. When the total volume increased from 4 mL to 10 and 20 mL, the size and PDI of the nanogels also increased significantly. Conversely, when the total volume decreased from 4 mL to 2 mL, the difference in size and PDI was insignificant. Interestingly, the ZP of the nanogels showed an opposite trend, where the ZP of the nanogels did not change with increasing total volume but increased with scaling down. No difference was observed between nanogels fabricated at 2 and 20 mL. However, the encapsulation efficiency increased when the total volume changed from 4 to 10 mL. In short, the results demonstrated that changing the volume pro-rata in nanogel fabrication also altered the nanogel properties, despite the use of an identical fabrication condition and formulation. Nanogel fabrication could not be scaled up by increasing the volume pro rata beyond the design space of the model, but it could be scaled down to 2 mL using the same condition without much disruption to the nanogel properties. Thus, scaling up the production of nanogels to a larger batch requires adjustments to the fabrication conditions.

## 4. Conclusions

The effect of various processing factors in the fabrication on the properties of propranolol-loaded nanogels was systematically evaluated using the Design of Experiment approach. Ten processing factors were evaluated in a definite screening design, with a total of 26 experiments performed. Hydrodynamic size, PDI, zeta potential, and encapsulation efficiency were determined as the dependent factors. The results demonstrated that the properties of the nanogels are greatly influenced by the fabrication process. The critical process parameters (CPPs) for the nanogels fabrication process included temperature, stirring rate, chitosan grade, crosslinker choice, the addition rate of the crosslinker, the volume of the glass vial, and total volume, which should be monitored and controlled to prepare nanogels with the desired properties. Moreover, these parameters should also be reported to ensure the reproducibility of the nanogels. It is noted that a limitation of this study is that it is not possible to determine the effect of chitosan grade due to the degree of deacetylation, molecular weight, or a combination effect. Thus, using chitosan from a different manufacturer or batch could produce nanogels with very distinct properties. With the use of response surface methodology, the optimal fabrication for nanogels utilised MMW chitosan and TPP. The addition rate for TPP solution was set at 2 mL/min, while the solution was then stirred at a temperature of 50 °C with a stirring speed of 600 rpm. The volume of the glass vial used was 28 mL, while the stirrer size was 20 mm. Furthermore, scaling up to the optimal condition of the nanogels was also investigated in this study. Size and PDI were found to increase significantly when the total volumes were beyond the design space of the model, despite other processing factors being kept unchanged. Thus, the result elucidated that the fabrication method could not be scaled up by increasing the volume pro rata; hence, adjustments to the fabrication conditions are required to upscale a larger batch production.

## Figures and Tables

**Table 1:** Dimensions of various stirrers and glass vials that were used in the definitive screening design.

**Stirrer**	**Length (mm)**	**Width (mm)**	**Thickness (mm)**
Small	12.8 ± 0.0	4.8 ± 0.0	4.7 ± 0.1
Medium	15.1 ± 0.1	4.8 ± 0.0	4.6 ± 0.0
Large	19.9 ± 0.0	6.0 ± 0.0	6.2 ± 0.3
**Glass Vial Volume (mL)**	**External Diameter (mm)**	**Height (mm)**	**Glass Wall Thickness (mm)**
14	23.18 ± 0.1	58.37 ± 0.1	1.05 ± 0.0
28	27.46 ± 0.0	71.69 ± 0.2	1.09 ± 0.0

**Table 2:** Processing factors in the nanogels fabrication and dependent variable and the experimental design matrix of DSD design.

B	T (°C)	TV (mL)	SR (rpm)	AR (mL/min)	D (h)	CS	CR	GV (mL)	SS (mm)	Size (nm)	PDI	ZP (mV)	EE (%)
1	25	10	300	0.5	1	MMW	TPP	14	20	130.4	0.235	33.1	28.8%
2	25	7	300	2	1	LMW	TPP	28	12	129.5	0.266	17.01	31.4%
1	25	10	300	2	1.5	MMW	Pyro	14	12	240.9	0.29	30.1	34.0%
1	25	4	300	2	2	LMW	TPP	14	20	139.6	0.257	38.84	33.2%
2	25	4	300	0.5	2	MMW	Pyro	28	20	195.2	0.282	35.9	38.3%
1	25	10	450	0.5	2	LMW	Pyro	28	12	158.3	0.282	31.74	37.0%
1	25	10	600	2	1	LMW	Pyro	28	20	148.7	0.275	25.02	33.1%
2	25	4	600	1.25	1	LMW	Pyro	14	20	99.2	0.224	26.36	29.7%
2	25	4	600	0.5	1	MMW	TPP	28	12	152.2	0.261	35.76	36.0%
2	25	4	600	2	2	MMW	Pyro	14	12	163.7	0.301	29.4	29.8%
2	25	10	600	0.5	2	LMW	TPP	14	16	199.1	0.318	38.47	16.6%
1	37.5	4	300	0.5	1	LMW	Pyro	14	12	153.7	0.289	25.29	35.1%
1	37.5	7	450	1.25	1.5	MMW	TPP	28	16	142.6	0.237	36.09	28.7%
1	37.5	7	450	1.25	1.5	LMW	Pyro	14	16	105.5	0.258	24.8	31.0%
1	37.5	10	600	2	2	MMW	TPP	28	20	203.9	0.303	24.29	28.8%
2	50	10	300	0.5	1	LMW	TPP	28	20	255.5	0.355	33.69	29.9%
2	50	4	300	2	1	MMW	Pyro	28	16	155.1	0.272	37.39	34.7%
1	50	4	300	0.5	2	MMW	TPP	14	12	146	0.282	30.81	32.4%
2	50	10	300	1.25	2	MMW	TPP	28	12	170.8	0.258	33.58	29.6%
2	50	10	300	2	2	LMW	Pyro	14	20	138.8	0.269	32.69	31.1%
1	50	4	450	2	1	MMW	TPP	14	20	110	0.218	28.44	35.7%
1	50	10	600	0.5	1	MMW	Pyro	28	12	100	0.244	22.03	33.2%
2	50	10	600	2	1	LMW	TPP	14	12	107.3	0.229	19.73	31.0%
1	50	4	600	0.5	1.5	LMW	TPP	28	20	175	0.243	26.18	41.9%
1	50	4	600	2	2	LMW	Pyro	28	12	161.5	0.292	31.81	31.1%
2	50	7	600	0.5	2	MMW	Pyro	14	20	112.5	0.254	28.34	40.7%

Keys: blocking (B), temperature (T), total volume (TV), stirring speed (SR), the addition rate of TPP solution (AR), duration of stirring (D), chitosan grade (CS), choice of crosslinker (CR), glass vial size (GV), size of the magnetic stirrer (SS), low molecular weight chitosan (LMW), medium molecular weight chitosan (MMW), sodium triphosphate (TPP) and pyrophosphate (Pyro).

**Table 3:** ANOVA and lack of fit test results for the DSD model for various independent variables.

Independent Variables	Source of Variations	Degree of Freedom	Sum of Squared	Mean Squares	F Value	Prob. > F	Significance
Size	Model	5	37,432.870	7486.570	43.225	<0.0001	Significant
	T	1	2526.010		14.584	0.0011	Significant
	SR	1	2180.853		12.591	0.0020	Significant
	CS	1	16,225.800		93.682	<0.0001	Significant
	CR	1	16,416.877		94.785	<0.0001	Significant
	T × CS	1	1978.637		11.424	0.0030	Significant
	Residual	20	3764.03	173.20			
	Lack of fit	14	2848.699	203.478	1.984	0.2046	Not significant
	Pure error	6	615.334	102.556			
PDI	Model	2	0.013	0.007	14.076	0.0001	Significant
	T	1	0.006		12.274		Significant
	CR	1	0.006		13.526		Significant
	Residual	23	0.011	0.000			
	Lack of fit	3	0.000	0.000	0.062	0.9764	Not significant
	Pure error	20	0.011	0.001			
EE	Model	2	0.0024	0.012	8.119	0.0021	Significant
	AR	1	0.007		4.727	0.0402	Significant
	GV	1	0.019		12.692	0.0017	Significant
	Residual	23	0.0341	0.001			
	Lack of fit	3	0.003	0.001	0.5524	0.6524	Not significant
	Pure error	20	0.031	0.002			
ZP	Model	2	421.314	210.657	11.650	0.0003	Significant
	CS	1	373.803		20.673	0.0001	Significant
	SS	1	72.002		3.982	0.05810	Not significant
	Residual	23	415.884	18.082			
	Lack of fit	3	29.797	9.932	0.5145	0.6770	Not significant
	Pure error	20	386.087	19.304			

Keys: T is temperature, SR is stirring rate, CS is chitosan grade, CR is crosslinker choice, AR is addition rate, GV is glass vial volume and SS is stirrer size.

## Data Availability

Data are contained within the article.
